# The influence of family cohesion on self-regulation and anxiety problems among African American emerging adults

**DOI:** 10.1371/journal.pone.0261687

**Published:** 2022-01-21

**Authors:** Danielle A. Augustine, Kalsea J. Koss, Emilie P. Smith, Steven M. Kogan

**Affiliations:** 1 Department of Human Development and Family Science, University of Georgia, Athens, Georgia, United States of America; 2 Department of Human Development and Family Studies, Michigan State University, East Lansing, Michigan, United States of America; University of the West Indies at Saint Augustine, TRINIDAD AND TOBAGO

## Abstract

Although African Americans have lower rates of anxiety in childhood than other racial and ethnic minority groups, they seem to experience escalating rates during emerging adulthood. Despite this, few studies have examined factors associated with anxiety during emerging adulthood among African American populations. The current study investigated the extent to which late adolescent family relationships affect anxiety problems among African American emerging adults. Informed by family development theory, family cohesion was hypothesized to indirectly effect anxiety problems through self-regulation. This model was tested with three waves of data (ages 17, 19, 21) from African Americans participating in the Maryland Adolescent Development in Context Study. Study findings were consistent with the hypothesized model: family cohesion forecasted decreased anxiety problems, indirectly, via increased self-regulation. This finding suggests that families may be an important promotive process for anxiety problems during emerging adulthood. Prevention programs that target family processes may be able to reduce anxiety problems in emerging adult African Americans.

## Introduction

Anxiety is characterized by heightened, relentless fears and worries about everyday events [1]. Clinical anxiety disorders are prevalent in the United States, affecting a third of Americans at some point in their life [2]. There are many individuals whose anxiety is not debilitating enough for a clinical diagnosis, but still experience anxiety symptoms such as nervousness, restlessness, excessive worry, sense of dread, increased heart rate, and sleep problems [3, 4]. These individuals are said to have subclinical anxiety, and, because they do not have clinical anxiety, may not be represented in the prevalence rates of anxiety [5]. To understand how anxiety unfolds, it is important to study those with subclinical anxiety problems in addition to those with clinical anxiety. Most anxiety problems manifest prior to age 30 [6]. Some studies suggest that emerging adulthood (~ages 18–25) is a developmental phase when anxiety problems are particularly likely to start or escalate in severity [7–9]. This time of life can be both stressful as well as rewarding because of multiple role transitions [6, 9, 10]. Between the ages of 18 and 25, many adults leave childhood homes, pursue post-secondary education, start full-time jobs, and enter serious romantic relationships. These transitions may be stressful for some and lead to anxiety problems [11]. Approximately 22% of emerging adults will experience anxiety problems [11, 12].

Racial and ethnic group differences in anxiety rates and severity have been observed. Although African Americans have lower rates of anxiety in childhood than other racial and ethnic minority groups, they seem to experience escalating rates during emerging adulthood [13, 14]. Moreover, when African Americans do experience anxiety, it is often more chronic and severe than their peers from other racial groups [13]. Little research, however, has investigated anxiety among African American emerging adults. Of particular importance, no studies, to our knowledge, examine contextual factors that are associated with reductions in anxiety as African American youth transition into adulthood. Family development theory suggests that families are an important context that may influence mental health outcomes [15]. In particular, this perspective underscores the importance of *family cohesion* in promoting the development of youths’ autonomy and their ability to navigate new contexts.

Family cohesion describes nurturant communication, warmth, emotional support, and involvement between family members [16–18]. In general, family cohesion improves during the transition to emerging adulthood [19]. Many emerging adults report warmer, closer relationships with their parents than they did in adolescence. Emotional support also increases during this period; many emerging adults turn to their parents for sympathy, advice, and help [20]. Per family development perspectives, cohesive family relationships act as an emotional safety net and help prevent anxiety problems during emerging adulthood [21–23]. Data with adolescents support this link: youth from cohesive families report better psychological outcomes and, by extension, fewer anxiety problems, than youth from less cohesive families [24, 25]. For example, youth who attend college may continue to rely on their family for stress management [26, 27]. Families help emerging adults manage their stress by talking to them and providing emotional support. This support can help to alleviate stress, thus reducing anxiety problems [26, 28].

Although these studies suggest that cohesive families may reduce poor psychological outcomes in emerging adulthood, the extent to which family cohesion is associated with anxiety problems among African American emerging adults is not well studied, despite close family ties being culturally important for many African American families [29]. Data on African American children and adolescents suggest that close family relationships may reduce anxiety problems in some youth [30–32]. This link has also been found in middle-age adult samples [33, 34], which suggests that families may be an important promotive factor for anxiety problems throughout the life course. Thus, we hypothesized that family cohesion in late adolescence would be associated with decreased anxiety problems during emerging adulthood.

We further hypothesized that self-regulation may be a mechanism through which family cohesion affects anxiety problems during emerging adulthood. Self-regulation refers to individuals’ ability to control their emotions, behaviors, and thoughts [35]. Emerging data suggest that self-regulation may be a promotive factor of anxiety problems in African American adults [36], but the role of families in the development of self-regulation in African American emerging adults is understudied. Studies with children and adolescents provide some insight into how families, self-regulation, and anxiety problems may be related. Among children, families play a key role in shaping children’s “emotional worlds.” Warm, sensitive families who provide children with structures, rules, and strategies to help manage their emotions support the development of self-regulation [37]. In turn, highly regulated children are able to manage negative emotions such as worry, sadness, and anger which makes them less vulnerable to anxiety problems [38]. Data suggests that the association between families, self-regulation, and anxiety problems may persist into adolescence and emerging adulthood [25, 39–41]. The continued influence of families on emerging adults’ self-regulation and anxiety problems is plausible because (a) neurocognitive systems associated with self-regulation continue to develop until a person’s mid-twenties as the prefrontal cortex continues maturing and (b) this development is affected by contextual influences [42, 43]. These changes suggest that development of self-regulation is ongoing during emerging adulthood and that young people may continue to need emotional and instrumental support and close family ties for optimal self-regulation and mental health, particularly when they experience times of trouble or stress [44].

Taken together, theory and extant research suggest that cohesive families may act as an emotional safety net which supports the development of self-regulation during emerging adulthood. With this safety net in place, emerging adults may feel comfortable exploring new opportunities while navigating the potential emotional turmoil of this challenging developmental period. The extent to which family cohesion forecasts self-regulation and reduced anxiety problems among African Americans emerging adults is not well studied. The current study addresses this gap. Informed by family development perspectives and previous research, family cohesion is hypothesized to indirectly effect anxiety problems through self-regulation. This hypothesis was tested via a secondary analysis of three waves of data from African Americans participating in the MADICS Study of Adolescent Development in Multiple Contexts (MADICS) controlling for antecedent family support.

## Methods

### Participants

Youth and their families were recruited from 23 middle schools in Prince George’s County, Maryland [45, 46]. Prince George’s County is a diverse, wealthy county located near Washington, D.C. Prince George’s County had a large middle-class African American community when MADICS started in 1990; the median household income for African Americans was $41,265, while the national average was $18,676 [45, 46]. At baseline (Wave 1) in 1991, 224 African Americans participated in the study. Of these youth, 52.7% were male and 47.3% were female. At Wave 1, 29.0% of parents earned less than $25,000; 44.0% of parents earned between $25,000 and $50,000; 20.0% of parents earned between $50,000 and $75,000; 7.0% and of parents earned more than $75,000. At Wave 4, 886 African American youth in eleventh grade participated in the study in 1996. Of these participants, 51.0% were male and 49.0% were female. At Wave 4, 15.2% of parents earned less than $25,000; 30.5% of parents earned between $25,000 and $50,000; 28.0% of parents earned between $50,000 and $75,000; and 33.2% of parents earned more than $75,000. At Wave 5, 358 (40%) African American youth participated in the study in 1998, a year after they graduate high school. Of these participants, 42.0% were male and 58.0% were female. A number of participants were not retained after Wave 4 due to the use of mailed surveys at Wave 5 instead of interviews. At Wave 6, 369 (42%) African American youth participated in the study in 2000, three years after they graduated high school. Of these, 42.1% were male and 57.9% were female. Attrition analysis investigated if family income, sex, self-regulation, and family cohesion (a composite of 4 family scales described below) were associated with retention status at W6. Results indicated that family income, self-regulation, and family cohesion were not associated with retention status at W6. Only sex was related participation in Wave 6.

### Procedure

The MADICS was a longitudinal study investigating psychological and behavioral determinants of developmental trajectories of youth living in Prince George’s County, Maryland, a county located near Washington, D.C. [45, 46]. MADICS is one of the few longitudinal studies investigating the developmental outcomes of a predominantly African American sample from adolescence to emerging adulthood [46]. Data were collected over eight waves spanning adolescence and young adulthood. Given the focus on emerging adulthood, the current study conducted a secondary analysis of data from African American youth from Waves 4–6 (ages 17, 19, 21) of the study. This study also uses a latent variable created from measures of family cohesion provided in middle adolescence as a covariate (Wave 3, age 15). During Waves 3 and 4 of MADICS, trained interviewers administered surveys to youth and their caregivers in their homes [45, 46]. Youth and their caregivers received $20 each for their participation. Only young people completed self-administered surveys during Waves 5 and 6. During Waves 5 and 6, surveys were mailed to participants. Youth received $35 for their participation at these waves [45, 46]. The current study was reviewed by the Human Subjects Office at the University of Georgia. It was determined that secondary analysis of non-identifiable data does not constitute research with human subjects.

### Instruments

#### Family cohesion

Family cohesion was assessed with four self-report scales: family emotional support, supportive communication with parents, closeness with parents, and closeness with family members. Family emotional support was evaluated at Waves 3 and 4, using four items from a scale adapted from the Philadelphia Family Management Study [47]. Youth self-reported how much support they receive from their family members using a 5-point Likert scale [45]. A sample item from the scale is: “How often do your family members support each other?” Cronbach’s alpha for Wave 3 was .79 and Wave 4 was .81. Supportive communication with parents was measured at Waves 3 and 4 using four items derived from the Michigan Study of Adolescent Life Transitions study (MSALT) [48]. Youth self-reported the frequency of supportive communication with their parents using a 5-point Likert scale [45]. A sample item from the scale is: “how often do you talk with your parent about problems you are having in school?” Cronbach’s alpha at Waves 3 was .78 and Wave 4 was .82. Closeness with parents was measured at Waves 3 and 4 using three items from the Iowa Youth and Family Study [49, 50]. In the closeness with parents scale, youth self-reported how close they are to their parents using a 4-point Likert scale [45]. An example is: “how close do you feel to your parent or current guardian?” Closeness with parents had acceptable internal consistency at Waves 3 (*α* = .69) and Wave 4 (*α* = .68). Closeness with family members at Waves 3 and 4 were measured using five items from the Iowa Youth and Family Study [49, 50]. Youth self-reported how close they are to their family members using a 6-point or 4-point Likert scale. An example of an item for the closeness with family members is: “How important is it to your family that you all do things together on weekends?” Items were standardized and summed to index family closeness. The resulting measure had reasonable internal consistency at Wave 3 (*α* = .72) and at Wave 4 (*α* = .74). All scales were used to create a latent variable of family cohesion.

#### Self-regulation

Self-regulation at Waves 4 and 5 was measured using eleven items from the Philadelphia Family Management Study [47]. Emerging adults self-reported their self-regulation using a 5-point Likert scale [45]. A sample item from the self-regulation scale is: “How often can you find a way to solve a problem, even when others get discouraged?” Self-regulation evinced acceptable internal consistency at Wave 4 (*α* = .73) and good internal consistency at Wave 5 (*α* = .85). Items were summed to create an index of self-regulation skills.

#### Anxiety problems

Anxiety problems at Waves 5 and 6 were evaluated using the 8-item Anxiety subscale of the Symptom Checklist-90-Revised (SCL-90-R) [51]. Emerging adults self-reported how often they experienced anxiety symptoms such as excessive worry using a 6-point Likert scale [45]. An example item is: “During the past 12 months, how often have you felt worried about things that were not likely to happen?” Anxiety problems has good internal consistency at Wave 5 (*α* = .83) and Wave 6 (*α* = .88). Items were summed to create an index of anxiety problems.

#### Sociodemographic variables

Youth reported their sex at Wave 4. Sex was coded as 1 (*male*) or 2 (*female*). Parents reported their household income at Wave 4 using the item, “from all sources of income, tell me your total family income before taxes in 1995.” Responses were coded in $5,000 increments up to $200,000 or more, ranging from 1 (*less than $5*,*000*) to 25 (*more than $200*,*000*).

### Analytic plan

Study hypotheses were tested with structural equation modeling as implemented in Mplus [52]. Structural equation modeling facilitates mediation analysis and allows researchers to test hypotheses while accounting for error by using latent constructs [53, 54]. Latent variables are unobserved variables created from several observed variables. In the current study, latent factors for family cohesion at Waves 3 and 4, were created using 4 scales: family emotional support, supportive communication with parents, closeness with parents, and closeness with family. The latent constructs were first evaluated with a confirmatory factor analysis prior to testing study hypotheses in a structural model.

A structural model, specifically an indirect effects model, was specified to test the proposed hypothesis, following the recommendations by Little [55] and Kline [53] for controlling antecedent repeated measures. We controlled for antecedent family cohesion (Wave 3) in order to better isolate the effect of late adolescence family cohesion (Wave 4) on self-regulation and anxiety problems in emerging adulthood. We also controlled for self-regulation at Wave 4 in order to index the effect of family cohesion on changes in self-regulation from Wave 4 to Wave 5. Similarly, anxiety at Wave 5 was controlled to index changes from Wave 5 to Wave 6. We controlled for sex and family income in all structural models. Significance of the indirect effect was tested using bias-corrected 95% confidence intervals procured from bootstrapping [54, 56]. MacKinnon et al. [57] recommends using bootstrapping to examine indirect effects because it enables researchers to simultaneously estimate the entire model, instead of in a step-wise fashion. Bootstrapping estimated the indirect effects of family cohesion on anxiety problems via self-regulation using 1,000 bootstrap resamples [54]. Missing data were handled using full information maximum likelihood [58]. Model fit was evaluated using the root mean square error of approximation (RMSEA), standardized root mean square residual (SRMR), comparative fit index (CFI), and the Tucker Lewis Index (TLI) [53, 54]. Model fit was considered good if RMSEA was less than .08, SRMR was less than .08, CFI was greater than .90, and TLI was greater than .90.

## Results

**Fig 1:**
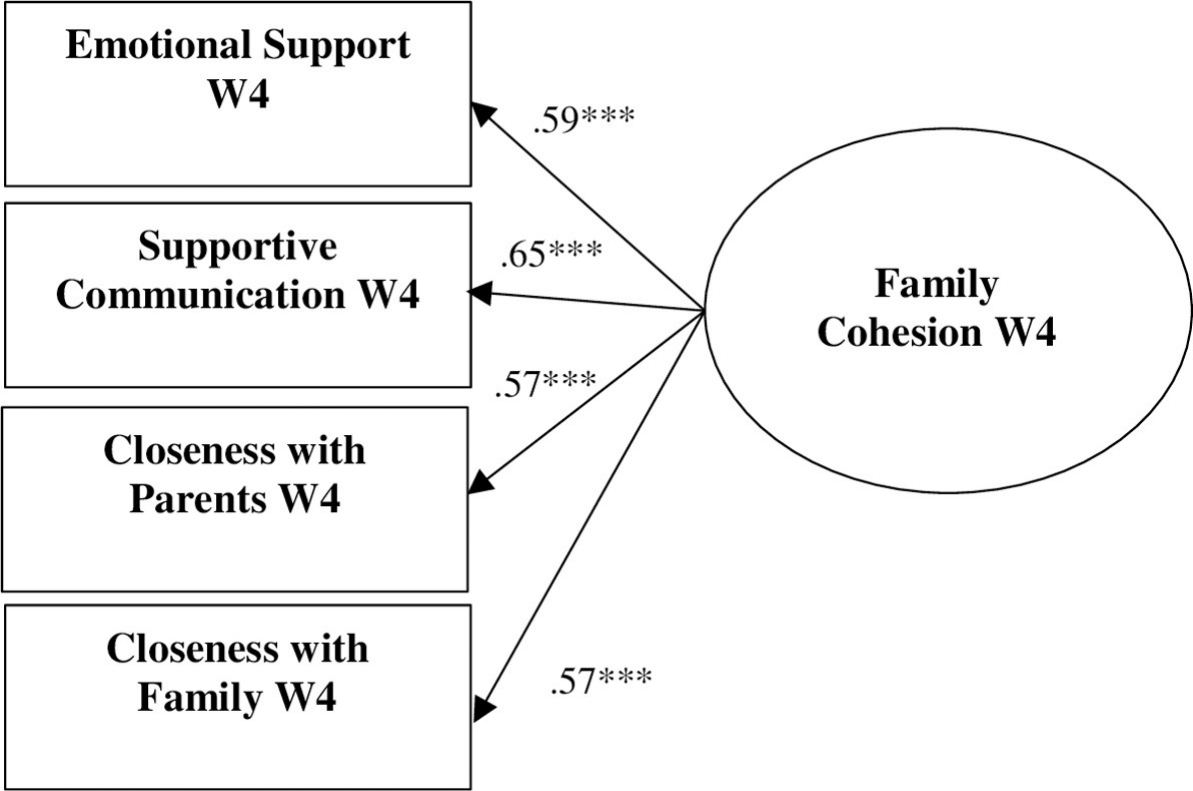
Measurement model for family cohesion W4. Standardized results are presented. * *p* < .05 (two-tailed). ** *p* < .01 (two-tailed). ***p < .001 (two-tailed).

**Fig 2:**
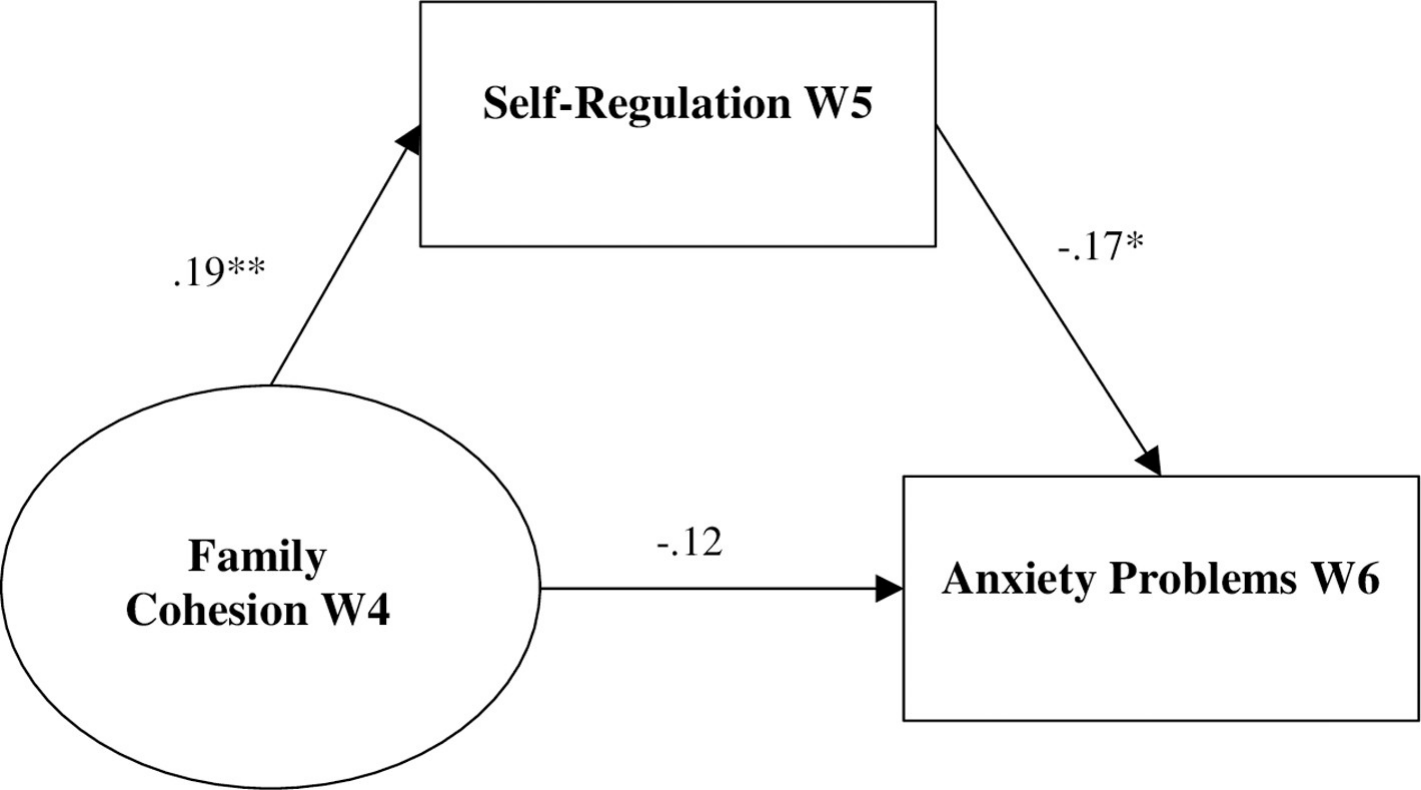
Indirect effects model. Standardized results are presented. Family cohesion at W3, self-regulation at W4, and anxiety problems at W5 were controlled for in the model but removed from the figure for clarity. * *p* < .05 (two-tailed). ** *p* < .01 (two-tailed). ***p < .001 (two-tailed).

Descriptive statistics and bivariate correlations for the 886 African Americans participating in Wave 4 of MADICS are presented in Table 1. The measurement model for family cohesion Wave 4 (see Fig 1) fit the data well: χ^2^ (2) = 1.57, *p* = .46, RMSEA = .00, SRMR = .01, CFI = 1.00, TLI = 1.00. All indicators fit as expected and were significant (*p* < .001). The initial model testing study hypotheses did not fit the data well (χ^2^ (60) = 190.73, *p* < .001, RMSEA = .05, SRMR = .05, CFI = .91, TLI = .87), so sex and family income were removed post hoc. Fig 2 depicts the results of our test of the study hypotheses; S1 Table presents all estimated parameters. The indirect effect model fit the data well: χ (46) = 108.69, *p* < .001, RMSEA = .04, SRMR = .04, CFI = .95, TLI = .93. After controlling for W3 family emotional support, W4 self-regulation, and W5 anxiety problems, family cohesion W4 was associated with self-regulation W5, which in turn was related to anxiety problems W6. A 95% confidence interval [-.19, -.01] indicated that the indirect effect of family cohesion on anxiety problems through self-regulation was significant.

**Table 1:** Descriptive statistics and bivariate correlations.

	1	2	3	4	5	6	7	8	9	10	11	12	13	14
1. Family Emotional Support W3	1													
2. Family Emotional Support W4	.38**	1												
3. Supportive Parental Communication W3	.32**	.26**	1											
4. Supportive Parental Communication W4	.23**	.37**	.38**	1										
5. Closeness with Parents W3	.36**	.32**	.51**	.25**	1									
6. Closeness with Parents W4	.25**	.36**	.30**	.37**	.51**	1								
7. Closeness with Family W3	.38**	.26**	.39**	.12**	.42**	.24**	1							
8. Closeness with Family W4	.24**	.33**	.24**	.39**	.27**	.31**	.46**	1						
9. Self-Regulation W4	.17**	.25**	.06	.16**	.18**	.17**	.06	.05	1					
10. Self-Regulation W5	.23*	.13*	.07	.16*	.19**	.15**	.16**	.17**	.39**	1				
11. Anxiety W5	-.09	-.04	-.01	.05	-.08	-.12*	-.11	-.08	-.12*	-.16**	1			
12. Anxiety W6	-.08	-.15*	-.03	-.07	-.17**	-.21**	-.12	-.20**	-.15*	-.29**	.48**	1		
13. Sex	.04	.03	.09	.22**	-.09*	-.10**	-.07	-.01	-.09*	-.03	.08	.10	1	
14. Family Income	.11*	.12*	-.02	-.04	.08	.01	0.6	-.01	.11**	.05	-.10	.04	-.04	1
Mean	4.00	3.97	3.48	2.60	3.25	3.26	3.86	2.37	3.97	3.80	2.42	2.52	1.49	11.74
SD	.75	.80	1.18	.75	.62	.62	.88	.65	.66	.64	.96	1.03	.50	5.73

Sex was coded as 1 (male) and 2 (female). Family income was coded in $5,000 increments up to $200,000 or more.

* *p* < .05 (two-tailed).

** *p* < .01 (two-tailed).

## Discussion

Informed by family development perspectives on emerging adult mental health, this study investigated the effects of family cohesion during late adolescence on self-regulation and anxiety problems as African Americans transition into emerging adulthood. Three key findings emerged. First, family cohesion in late adolescence was significantly related to increased self-regulation in emerging adulthood. Second, self-regulation was significantly related to reduced anxiety problems in emerging adulthood. Third, family cohesion in late adolescence indirectly effected anxiety problems in emerging adulthood through self-regulation.

The first finding suggests that family cohesion during late adolescence is an important process for supporting self-regulation during emerging adulthood. This finding is consistent with and extends previous research on family effects among children and adolescents [24]. In addition, there is experimental evidence that changing family processes can result in increased self-regulation among youth [25]. Less research has examined this link among emerging adult African Americans. Fosco et al. [41] found that cohesive families were associated with increased self-regulation in a sample of primarily White emerging adults. The current study is consistent with an extends Fosco et al’s [41] findings to an African American sample. This finding also is consistent with family development theory, which proposes that families reshape and refined relationships as members transition into new developmental stages [15]. According to this theory, some families will be able to manage the stress associated with the transition and continue to maintain their closeness; others may find the transition overwhelming, may experience a decline in cohesion, and may experience anxiety problems. Based on this theory and in light of the current findings, it is possible that cohesive African American families who support self-regulation in emerging adults may perceive the transition as manageable and reduce anxiety problems in emerging adults.

Our findings suggest that self-regulation is an important factor in reducing anxiety problems among emerging adults. This is in line with similar findings in investigations of children and emerging adults. For example, a study of children between the ages of 8 to 12 found that highly regulated children were able to manage negative emotions such as worry, sadness, and anger, which suggests that self-regulation may be an important promotive factor for anxiety problems [38]. Similar patterns were found in a study of female undergraduate students attending a university in the Southeastern United States. In this study, highly regulated college students reported few anxiety problems [59]. The current study supports these findings by documenting reduced anxiety problems in highly regulated African American emerging adults.

The indirect effect finding suggests that self-regulation may act as a mechanism through which family cohesion during late adolescence affects downstream anxiety. Similar results were reported by Brody and Ge [24] in a study of African American youth. Brody et al. [24] found that harsh parenting affected psychological adjustment indirectly via decrements in self-regulation. In contrast, our findings suggest that positive parenting may be a promotive factor for increased self-regulation which carries forward to affect fewer anxiety problems in emerging adulthood.

### Implications for prevention

Study findings have several implications for prevention. Prevention programs working to reduce anxiety problems in African Americans prior to and during the transition to adulthood should consider targeting enhancing family cohesion. The Adults in the Making (AIM) prevention program is an example of a program that seeks to increase family cohesion in order to improve emerging adult outcomes [60]. Families attending the program learned how to provide developmentally appropriate emotional support as adolescents transition into adulthood and begin taking more responsibility for their lives. Youth attending the program learned self-regulatory skills, such as self-control and coping skills. Family cohesion and self-regulatory skills developed through AIM were associated with decrease alcohol and substance use [60]. This finding suggests that prevention programs for emerging adults that target families and youth’s self-regulation may be effective in reducing poor mental health outcomes during the transition to adulthood.

### Limitations and future directions

The current study has several limitations. First, the sample comprised of African American youth living in Maryland, so these findings may not generalize to African American youth living in other cities or regions of the United States. Second, these findings are correlational. Experimental designs using preventive interventions [61] could be used in future studies to confirm the causal influence of family cohesion on self-regulation and anxiety problems. Third, participants self-reported family cohesion, self-regulation, and anxiety problems. Self-report measures are prone for biases from social desirability, which occurs when participants report a mix of what is socially desirable and the truth [62]. Self-report measures are also prone for recall bias because participants were asked to recall information about their families, self-regulatory processes, and anxiety problems over the past year [63]. Asking participants to recall information from a sizeable length of time may have affected the accuracy of the information reported by participants which may attenuate the magnitude of the relationships studied. It is possible that some participants may have under-reported or over-reported family cohesion, self-regulation, and anxiety problems. Self-report are also prone to common method bias, which occurs when constructs are assessed using the same measure and may cause stronger correlations between variables [64]. Future studies can prevent these biases by using multiple types of data (e.g., observational). Fourth, data on anxiety problems were not collected before Wave 5, when youth were aged 19. Future studies should collect data on anxiety problems prior to emerging adulthood in order to identify African Americans with pre-existing anxiety problems and those whose anxiety problems started in emerging adulthood. The trajectory of anxiety problems for these groups may look different and could affect how family cohesion and self-regulation act as promotive processes of anxiety problems in emerging adulthood.

## Conclusion

In summary, family cohesion in late adolescence was indirectly related to changes in anxiety problems in emerging adulthood through self-regulation. This finding suggests that families continue to serve as important promotive processes for self-regulation and anxiety problems during the transition to emerging adulthood. Prevention programs that incorporate the family may be able to reduce anxiety problems in emerging adult African Americans.

## Supporting information

S1 Table Unstandardized, standardized, and significance levels for the models. (DOCX)
