# Supplementary material for: The influence of family cohesion on self-regulation and anxiety problems among African American emerging adults
Source: PLoS One. 2022 Jan 21;17(1):e0261687. doi: 10.1371/journal.pone.0261687 (PMC8782322; doi:10.1371/journal.pone.0261687)
Supplement: S1 Table — (DOCX) [file pone.0261687.s001.docx]

| **S1 Table. Unstandardized, Standardized, and Significance Levels for the Measurement and Structural Models** | | | | |
| --- | --- | --- | --- | --- |
| Parameter Estimates | | Unstandardized Estimate (SE) | Standardized Estimate | *p* |
| Measurement Model for Family Cohesion W3 | |  |  |  |
|  | Family Cohesion W3 → Emotional Support W3 | 1.00*^f^* | .52 | --- |
|  | Family Cohesion W3 → Supportive Communication W3 | 2.05 (.21) | .67 | .000 |
|  | Family Cohesion W3 → Closeness with Parents W3 | 1.14 (.12) | .72 | .000 |
|  | Family Cohesion W3 → Closeness with Family W3 | 1.38 (.14) | .61 | .000 |
|  | Intercept for Emotional Support W3 | 4.00 (.03) | 5.36 | .000 |
|  | Intercept for Supportive Communication W3 | 3.48 (.05) | 2.95 | .000 |
|  | Intercept for Closeness with Parents W3 | 3.25 (.03) | 5.28 | .000 |
|  | Intercept for Closeness with Family W3 | 3.86 (.04) | 4.39 | .000 |
|  | Residual Variance for Emotional Support W3 | .41 (.03) | .73 | .000 |
|  | Residual Variance for Supportive Communication W3 | .77 (.06) | .55 | .000 |
|  | Residual Variance for Closeness with Parents W3 | .18 (.02) | .49 | .000 |
|  | Residual Variance for Closeness with Family W3 | .49 (.04) | .63 | .000 |
| Measurement Model for Family Cohesion W4 | |  |  |  |
|  | Family Cohesion W4 → Emotional Support W4 | 1.00*^f^* | .59 | --- |
|  | Family Cohesion W4 → Supportive Communication W4 | 1.04 (.11) | .65 | .000 |
|  | Family Cohesion W4 → Closeness with Parents W4 | .75 (.08) | .57 | .000 |
|  | Family Cohesion W4 → Closeness with Family W4 | .79 (.09) | .57 | .000 |
|  | Intercept for Emotional Support W4 | 3.97 (.03) | 4.96 | .000 |
|  | Intercept for Supportive Communication W4 | 2.60 (.03) | 3.48 | .000 |
|  | Intercept for Closeness with Parents W4 | 3.26 (.03) | 5.29 | .000 |
|  | Intercept for Closeness with Family W4 | 2.37 (.03) | 3.66 | .000 |
|  | Residual Variance for Emotional Support W4 | .42 (.03) | .66 | .000 |
|  | Residual Variance for Supportive Communication W4 | .32 (.03) | .58 | .000 |
|  | Residual Variance for Closeness with Parents W4 | .26 (.02) | .67 | .000 |
|  | Residual Variance for Closeness with Family W4 | .28 (.02) | .67 | .000 |
| Structural Model | |  |  |  |
|  | Family Cohesion W3 → Family Cohesion W4 | .82 (.10) | .70 | .000 |
|  | Self-Regulation W4 → Family Cohesion W4 | .12 (.03) | .16 | .000 |
|  | Family Cohesion W4 → Anxiety Problems W6 | -.24 (.14) | -.16 | .092 |
|  | Family Cohesion W4 → Self-Regulation W5 | .25 (.10) | .19 | .005 |
|  | Anxiety Problems W5 → Anxiety Problems W6 | .47 (.08) | .44 | .000 |
|  | Self-Regulation W5 → Anxiety Problems W6 | -.27 (.12) | -.17 | .033 |
|  | Self-Regulation W4 → Self-Regulation W5 | .34 (.06) | .35 | .000 |
|  | Covariance of Family Cohesion W3 and Anxiety Problems W5 | -.02 (.03) | -.06 | .000 |
|  | Covariance of Family Cohesion W3 with Self-Regulation W4 | .05 (.02) | .17 | .002 |
|  | Covariance Closeness with Family W3 with Closeness with Family W4 | .14 (.02) | .36 | .000 |
|  | Covariance Closeness with Parents W3 with Closeness with Parents W4 | .09 (.01) | .38 | .000 |
|  | Covariance Self-Regulation W4 with Anxiety Problems W5 | -.06 (.04) | -.10 | .109 |
|  | Mean for Anxiety Problems W5 | 2.44 (.05) | 2.55 | .000 |
|  | Mean for Self-Regulation W4 | 3.96 (.03) | 5.98 | .000 |
|  | Intercept for Anxiety Problems W6 | 2.50 (.51) | 2.46 | .008 |
|  | Intercept for Self-Regulation W5 | 2.33 (.28) | 3.61 | .000 |
|  | Variance for Anxiety W5 | .92 (.06) | 1.00 | .000 |
|  | Variance for Self-Regulation W4 | .44 (.02) | 1.00 | .000 |
|  | Variance for Family Cohesion W3 | .17 (.03) | 1.00 | .000 |
|  | Residual Variance for Anxiety Problems W6 | .76 (.09) | .74 | .000 |
|  | Residual Variance for Self-Regulation W5 | .34 (.03) | .81 | .000 |
|  | Residual Variance for Family Cohesion W4 | .11 (.02) | .46 | .000 |
| W5 Self-Regulation R-squared = .20 (*p* < .001); W6 Anxiety R-squared = .28 (*p* < .001). | | | | |
